# Preoperative Strength Training for Elderly Patients Awaiting Total Knee Arthroplasty

**DOI:** 10.1155/2014/462750

**Published:** 2014-02-13

**Authors:** D. M. van Leeuwen, C. J. de Ruiter, P. A. Nolte, A. de Haan

**Affiliations:** ^1^MOVE Research Institute Amsterdam, Faculty of Human Movement Sciences, VU University Amsterdam, Van der Boechorststraat 9, 1081 BT, Amsterdam, The Netherlands; ^2^Institute for Biomedical Research into Human Movement and Health, Manchester Metropolitan University, Manchester M1 5GD, UK; ^3^Department of Orthopedics, Spaarne Hospital, Spaarnepoort 1, 2134 TM Hoofddorp, The Netherlands

## Abstract

*Objective*. To investigate the feasibility and effects of additional preoperative high intensity strength training for patients awaiting total knee arthroplasty (TKA). *Design*. Clinical controlled trial. *Patients*. Twenty-two patients awaiting TKA. *Methods*. Patients were allocated to a standard training group or a group receiving standard training with additional progressive strength training for 6 weeks. Isometric knee extensor strength, voluntary activation, chair stand, 6-minute walk test (6MWT), and stair climbing were assessed before and after 6 weeks of training and 6 and 12 weeks after TKA. *Results*. For 3 of the 11 patients in the intensive strength group, training load had to be adjusted because of pain. For both groups combined, improvements in chair stand and 6MWT were observed before surgery, but intensive strength training was not more effective than standard training. Voluntary activation did not change before and after surgery, and postoperative recovery was not different between groups (*P* > 0.05). Knee extensor strength of the affected leg before surgery was significantly associated with 6-minute walk (*r* = 0.50) and the stair climb (*r* − = 0.58, *P* < 0.05). *Conclusion*. Intensive strength training was feasible for the majority of patients, but there were no indications that it is more effective than standard training to increase preoperative physical performance. This trial was registered with NTR2278.

## 1. Introduction

Knee osteoarthritis (OA) is a degenerative joint disease which is characterized by a gradual loss of cartilage [1] and can result in pain, limited physical performance, and lower quality of life [2]. If conservative treatment is ineffective, patients may decide to undergo a total knee arthroplasty (TKA), which can significantly reduce knee pain and can increase physical performance in patients with severe OA [1]. For patients undergoing TKA, the isometric strength of the knee extensors was shown to decrease by up to 60% four weeks after surgery, and this decrease was accompanied by decreases in the ability to voluntarily activate the knee extensor muscles [3]. Even after six months to thirteen years following TKA, the strength of the knee extensor muscles at involved side remains 12–30% lower than the uninvolved side, and strength almost never matched values for healthy controls [4]. This postoperative weakness has important consequences for activities of daily life, because knee extensor strength is strongly related to functional performance, such as walking and stair climbing [5] especially after TKA [6]. There are indications that preoperative strength is related to postoperative abilities [7, 8]. Intensive strength training *after* TKA has shown to be beneficial for decreasing pain and improving strength and physical performance when compared to usual care [2]. Multiple studies have investigated the effect of preoperative strength training on postoperative recovery [9–15]. However, few of these studies reported significant increases in preoperative strength following the training. Reviewing these studies, it is clear that the intensity of training, when documented, was either rather low [10, 13–15] or not progressively increased [13] or the number of sessions was too small to produce significant training effects [9]. Progressive, high intensity strength training is recommended to increase muscle strength [16]. Because the preoperative training period is typically rather short (the time between the decision for TKA and the actual surgery is typically 4 to 8 weeks), a high intensity and progressive loading may be needed to increase preoperative strength and performance and therefore promote postoperative recovery. However, it is unclear if this type of training is feasible in this patient group, since pain may be a limiting factor.

The aims of the present study were to investigate the feasibility and the effects of additional preoperative high intensity strength training for elderly patients awaiting TKA compared to standard preoperative training in a pilot study. We hypothesised that preoperative intensive strength training would lead to increases in strength and performance before surgery. We hypothesised that increases in strength were primarily caused by improved voluntary activation, because the first adaptations to strength training are primarily neural [17] and training time is limited.

## 2. Methods

### 2.1. Participants

All patients above 55 years awaiting TKA in the Spaarne Hospital in Hoofddorp were considered candidates for the present study and were asked to participate. Patients were excluded if they had (1) American Society of Anesthesiologists (ASA) score >2 [18], (2) contraindications for training the lower limbs, or (3) contraindications for electrical stimulation (unstable epilepsy, cancer, skin abnormalities, or having a pacemaker).

All patients had at least 1 year symptoms of severe osteoarthritis of the knee (Kellgren and Lawrence [19] grades 3 and 4). For additional exercise, patients were asked about their physical activity at each measurement occasion. The patients did not perform strength training before inclusion in this study. No severe coexisting diseases were present. Therefore, we do not expect a limiting effect on function or exercise responses of our participants.

### 2.2. Sample Size

Isometric knee extension strength of the surgical leg before TKA was defined as the primary outcome variable for the power analysis. The effect size for strength training with patients having osteoarthritis has been reported to be 0.35 [20] and 0.30 for preoperative training [14]. Because the control group also received therapy, we used an effect size of 0.20. For 0.8 power, *α* = 0.05 and assuming a correlation of 0.85 between repeated measurements, a total of 18 participants was needed to assess significant differences between groups over time. Because 4 participants dropped out before the second measurement, four additional patients were included and in total 22 patients were enrolled in the study.

### 2.3. Randomization and Blinding

Participants were randomized in a 1 : 1 ratio (parallel design) to either the standard treatment or standard treatment with additional strength training. A research nurse approached potential candidates by phone, generated the random allocation sequence with use of custom software, enrolled patients, and assigned them to the interventions. Randomization was done by minimization of gender and age (median age of patients on the waiting list). After the inclusion of 15 patients, 2 participants had dropped out and two patients received the intervention instead of standard training and the ratio between strength training and standard training was 10/3. To increase comparability between groups, the remaining 7 patients were allocated to the standard training group. The principal investigator (DL) was blinded during measurements, but not during analyses of the results. The participants and therapists were not blinded.

### 2.4. Surgical Procedure

Patients underwent an uncemented TKA (mobile bearing total knee prosthesis, LCS Complete, Depuy, Warsaw, Indiana, United States) with standardized perioperative protocol and the same surgical technique. The surgical technique consisted of a midline incision with a flexed knee, medial arthrotomy, and bone cuts with Milestone instruments without the use of tourniquet or drains. Perioperative antibiotics (Kefzol 1 gram i.v.) and antithrombotics (Fraxiparine 0.3 mL i.m.) were used. The patients were mobilized the first day postoperatively. On average the patients left the hospital the 4th postoperative day. The surgeries were performed by experienced orthopedic surgeons (>50 TKA per year) and patients received protocolized inpatient physical therapy. The VU Medical Centre Medical Ethics Committee and the local ethics committee of the Spaarne Hospital approved the study, and all participants signed informed consent and the rights of the subjects were protected.

### 2.5. Intervention

**Figure 1:**
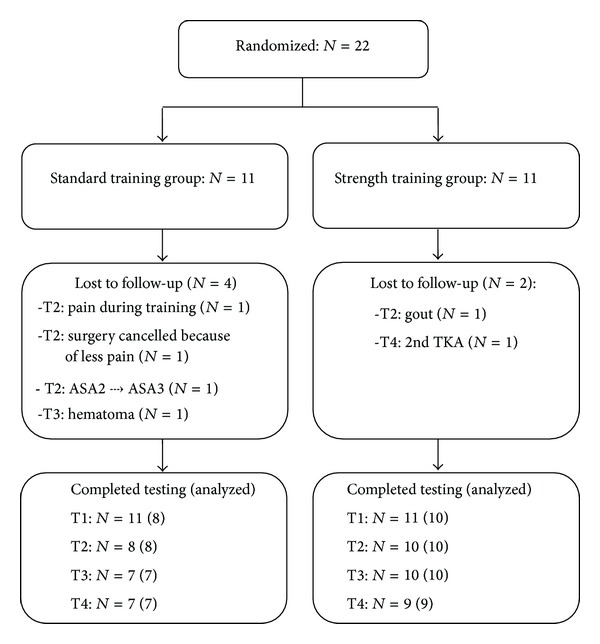
Flowchart of inclusion and follow-up in the two training groups.

Patients were allocated to standard treatment or received standard treatment with additional strength training (Figure 1). The standard training group received treatment according to guidelines from the Dutch association of orthopaedics [21] and the Dutch physiotherapy association (KNGF) [22] for training patients with OA. Therapy included information and advice, exercise of activities of daily life, training of walking with aids, maintenance of mobility, and aerobic training (walking, cycling), but the patients in this group were not allowed to perform resistance training. The intensive strength training group received the same treatment as the standard training group, with additional intensive strength training, consisting of a progressive strength program targeting the lower limbs.  Table 1 shows exercises, sets, and repetitions. We abstained from 1 RM testing to minimize pain sensations, because pain could lead to premature ending of the training. Instead, the training weights were adjusted to the abilities of the patients in relation to the number of repetitions. For the first training (3 × 15 repetition), patients were asked to perform the maximum number of repetitions with the selected weight. If either more or less than 15 repetitions were performed, the weight for the next set was adjusted with ~3% per repetition. For example, if a patient could perform 22 repetitions with 30 kg, the weight was increased with 7 (22 − 15 repetitions) ∗ 3% to 36.6 kg. Dumbbells or plates were used for small increments. To ensure progressive overload, repetitions decreased during the program, and the weights were increased when the number of repetitions decreased (~3% per repetition). For the squat exercise, intensity was increased by the increasing the range of motion before using dumbbells. Both the uninvolved and the involved limb were trained, and the weight was adjusted to abilities. The patients trained two to three times per week. In addition, a home program consisting of step-up and squat exercises was performed two to three times per week by the strength training group. In case of pain or other discomfort, the program was modified, but the intensity stayed as high as possible. After surgery, no interventions were applied; both groups received standard care including strength training. 13 physiotherapy centres participated by complying with the training program. 22 patients entered the study. Figure 1 shows allocation and follow-up.

### 2.6. Measures

All measurements were performed at the Spaarne Hospital before training (T1), after 6 weeks of training (T2, the week before TKA), 6 weeks after surgery (T3), and 12 weeks after surgery (T4).

#### 2.6.1. Feasibility

The feasibility was evaluated by checking training logs for adherence. Physiotherapists were instructed to note alterations of the training program. If training intensity was progressively increased and all exercises were executed, the program was considered feasible. The number and contents of the training sessions for the control group were also monitored by checking training logs.

#### 2.6.2. Torque Measurements 

Measurement of the contractile properties of the knee extensor and flexor muscles took place on a custom-made adjustable dynamometer. The lower leg was tightly strapped to a force transducer (KAP-E, 2 kN, A.S.T., Dresden, Germany), mounted to the frame of the chair about 25 cm distally of the knee joint. Participants sat in the dynamometer with a hip angle of 80° (0° is full extension), firmly attached to the seat with straps at the pelvis to prevent extension of the hip during contraction and a strap at the chest. All measurements were performed on both legs at a knee angle of 60° (0° is full extension), during isometric contraction. The nonsurgical leg was measured first to get accustomed to the procedures and electrical stimulation (see below). Force data were digitized (1 kHz), filtered with a 4th order bidirectional 150 Hz Butterworth low-pass filter, and stored on a PC for offline analysis. Force signals were corrected for gravity: the average force applied by the weight of the limb was set at zero. Torque was calculated by multiplying force with the distance between the force transducer and the knee joint. After 3 submaximal attempts, participants were asked to perform at least 3 maximal isometric knee extensions and flexions, and more if torque increased more than 10%, with at least two minutes of rest in between attempts. Maximal Voluntary Torque was defined as the highest torque recorded.

#### 2.6.3. Electrical Stimulation

Constant current electrical stimulation (pulse width 200 *μ*s) was applied through self-adhesive surface electrodes (Schwa-Medico, Leusden, The Netherlands) by a computer-controlled stimulator (model DS7A, Digitimer Ltd., Welwyn Garden City, UK). The distal electrode (8 × 13 cm) was placed over the medial part of the quadriceps muscle just above the patella and the proximal electrode (8 × 13 cm) over the lateral portion of the muscle to prevent inadvertent stimulation of the adductors. Before placing the electrodes the skin in the area of the electrodes was shaved. The stimulation current was increased until force in response to doublet stimulation (two pulses at 100 Hz) levelled off. After assessing maximal doublet force, the stimulation intensity was lowered and set to produce 50% of the maximal doublet force. This stimulation intensity ensured that a substantial amount of muscle mass was stimulated but significantly reduced discomfort at the same time [23]. Voluntary activation was calculated with use of the superimposed twitch technique. In short, upon a maximal voluntary contraction, a superimposed doublet was delivered to the muscle. Two seconds after each contraction, a (potentiated) doublet was delivered to the relaxed muscle to calculate voluntary activation with use of the following equation:

(1) Voluntary  activation  (%) =1−(superimposed  forcepotentiated  resting  doublet)∗100%

(see [23, 24]).

#### 2.6.4. Functional Tasks

A 5-time sit-to stand test was performed with the arms folded in front of the chest. Patients were instructed to stand up and sit down as quickly as possible. The six-minute walk test (6MWT) was used to quantify walking ability. Participants walked back and forth over 30 meters as many times as possible for a period of 6 minutes at their own pace, in a 60-meter-long corridor. The score recorded was the total distance travelled during 6 minutes. Participants were instructed to “walk as quickly and safely as you can for 6 minutes.”

To investigate stair-climbing, the time required to ascend 9 steps, turn around, and descend 9 steps was used. Participants were allowed to use the handrail and instructed to “walk as quickly and safely”. All tests except the 6MWT were repeated twice, and the fastest time was used for analysis. The 6MWT and the stair climb test are widely used as specific tests to quantify functional performance in patients [6, 25–28].

#### 2.6.5. Quality of Life and Physical Activity

Quality of life was assessed with the Western Ontario and McMaster Universities Osteoarthritis Index (WOMAC). The WOMAC questionnaire is used to obtain pain, stiffness, and functioning specifically for patients with OA. Scores were transformed to a 0 to 100 scale, where a 100 score signifies the best quality of life.

### 2.7. Statistics

Data are presented as mean ± SD. An ANOVA repeated measure was used to assess differences between the patient groups over time with a Bonferroni post-hoc correction. Two separate analyses were performed. The first analysis was done with preoperative data of patients with data on T1 and T2 (*N* = 18, T1 and T2) because the primary aim was to study effects of training on preoperative strength and performance. A second analysis was done on all complete data sets (T1–T4; *N* = 16) to investigate postoperative recovery (T3 and T4). Because not all patients were randomized, a per-protocol analysis was performed. A chi-square test was used to investigate differences in gender at baseline. Other baseline characteristics were analysed using the Kruskal-Wallis Test.

Effect size was calculated by subtracting the mean pre-post (T1-T2) change in the standard group from the mean pre-post change in the intensive training group, divided by the pooled pre-test standard deviation [29].

Pearson's correlation coefficient was used to investigate relationships between normally distributed variables. The level of significance for all tests was set at 0.05 and all analyses were performed with SPSS (version 16.0, SPSS Inc.).

## 3. Results

### 3.1. Feasibility

Twenty-two patients were recruited between October 2010 and December 2011. Figure 1 shows a flowchart of allocation and follow-up. All participants in the strength training group completed preoperative training, and there was one dropout in the standard training group. Four participants did not complete the 2nd preoperative test due to various reasons (Figure 1). Only data were analysed from patients who completed testing at T2.

Eight out of 11 patients in the strength training group completed training without adaptations. For 3 patients, small adjustments were made in intensity due to pain, to prevent premature ending of the training. Patients in the strength training group completed 12 ± 2 training sessions (range 11–17), and patients in the standard training group completed 11 ± 4 sessions (range 4–16).

In a pilot study, split squats were included in the training program, but too many patients reported pain during this exercise. Also reduction in range of motion in knee extensions and leg press showed to be an effective way to reduce pain, while maintaining a high training intensity.

Table 2 shows baseline characteristics for the patients in the strength training group, the standard training group who completed testing at T2, and the patients that dropped out. There were no significant differences between the groups.

### 3.2. Pre-Surgery Effects

#### 3.2.1. Strength Measures

Table 3 shows average values for strength measures. Before surgery there were no main effects of group or time: at baseline and T2, there were no significant differences in strength measures between groups and no changes in time for the total group. The effect size of maximal voluntary knee extension strength was 0.11. The post-hoc power was 0.87. There were also no significant interactions between group and time for any strength measure during this six-week preoperative training period. Strength training did not lead to increases in maximal knee extension torque (Table 3), voluntary activation, or doublet torque compared to the standard training group. At T1 and T2, the affected leg was not weaker than the unaffected leg and also voluntary activation was not different between both legs. The patients who dropped out before T2 did not have a significantly lower knee extension strength of the affected leg than the patients who completed testing at T2 (98 Nm versus 113 Nm, *P* = 0.61). The percentage of men and women in the two groups differed. Therefore, we also compared knee extension torque between the two groups at baseline with correction for gender. Both without (*P* = 0.929) and with correction for gender (*P* = 0.769), there was no difference in maximal knee extension torque at baseline.

#### 3.2.2. Functional Tasks

Before surgery (from T1 to T2) there were no main effects of “group,” but there were main effects of “time” for chair stand and 6MWT. For both groups combined chair stand (−1.1 s, *P* = 0.003) and 6MWT (25 m, *P* = 0.013) significantly improved before surgery (Table 3) and there was a trend for improvement in voluntary knee flexion strength of the affected side (3.4 Nm, *P* = 0.090). There were no significant interactions between “time” and “group,” indicating that any changes over time were similar between groups.

### 3.3. Post-Surgery Effects

#### 3.3.1. Strength Measures

After surgery there were no main effects of “group”. There was a main effect of “time” for maximal knee extension torque, doublet torque, and maximal knee flexion torque of the affected knee. Maximal torque of the knee extensors and doublet torque significantly decreased from T2 to T3 (6 weeks after surgery) and subsequently significantly increased from T3 to T4 (12 weeks after surgery, *P* < 0.05, Table 3). Knee flexor torque significantly increased from T3 to T4. At T4, maximal torque for knee extension and doublet torque were still between 20 and 30% lower compared with their preoperative values at T2, whereas maximal torque for knee flexion was back to baseline levels.

An unexpected finding was that there was a significant interaction between maximal torque of the knee flexors and group. Post-hoc testing indicated that maximal torque of the knee flexors decreased in the standard training compared to the intensive training groups between T2 and T3. As expected, doublet torque and knee extensor torque were lower for the affected side compared to the unaffected side on T3 and T4 and knee flexor torque was lower at T3 only (*P* < 0.05) compared to the unaffected side. Voluntary activation did not change after surgery.

#### 3.3.2. Functional Tasks

After surgery, there were no main effects of “group”, but there were main effects of “time” for several variables. Six weeks after surgery (T3), stair climbing time increased compared to T2 for both groups combined. From T3 to T4, significant main effects of time were present for chair stand, stair climb, 6MWT, and WOMAC score (*P* < 0.05, Table 3), without any significant interaction between group and time, again indicating that any changes over time were similar between groups.

### 3.4. Relationships between Quadriceps Strength and Physical Performance

Table 4 shows Pearson's correlation coefficients between maximal knee extension strength and chair, stair climb, and 6MWT performance at the four moments of testing. Only after surgery, maximal knee extension strength was related to chair stand (*r* ^2^ = 0.27 and *r* = 0.31, *P* < 0.05). Stair climb performance was related to maximal torque of both legs on all occasions (*r* ^2^ between 0.28 and 0.55, *P* < 0.05) and 6 MWT was significantly related to strength on T2, T3, and T4 (*r* ^2^ > 0.25, *P* < 0.05). In general, relationships between voluntary knee extensor strength and the functional tests became stronger over time.

## 4. Discussion

The main findings of the present study were that intensive strength training is feasible for the majority of the patients awaiting TKA, but that there are no indications that this intensive strength training is more effective than a standard training. The feasibility and pre- and postoperative effects will be separately discussed.

### 4.1. Feasibility

One of the aims of the present study was to investigate the feasibility of additional preoperative high intensity strength training for elderly patients awaiting TKA. In this training group, no patients dropped out because of the intervention. For 3 out of 11 patients, changes in the program had to be made because of pain or discomfort, but for the other 8 patients the training program could be performed without alterations. Although the groups were of limited size, intensive strength training seems feasible, at least for patients with ASA 1 or 2.

### 4.2. Pre-Surgery Effects

The effect size of the training on strength was small, 0.11, and not significant. This was not in line with our expectations, but it might be explained by the relatively short training time. Six weeks of training two times per week might not be enough to significantly increase strength in patients with end-stage OA, even if a high training intensity is used. In a systematic review investigating effects of strength training in OA patients, positive effects have been reported on strength, performance, and pain compared to control groups [30]. The average duration of the studies in this review was 9 months. Longer interventions may be needed to significantly increase preoperative strength and physical performance.

There were no differences in strength between the affected and the unaffected leg before surgery, although a difference in strength is often observed [14, 31, 32]. This might be explained by the fact that 2 patients were having a second TKA at a later stage and 4 patients already had an earlier TKA. This indicates that the nonsurgical leg was not “unaffected” in all patients.

The finding that strength training did not increase preoperative strength or promote postoperative outcome is in line with the majority of earlier studies [9, 10, 12–14]. In the present study, there were improvements in chair stand and the 6MWT for the entire group before surgery. It is important to note that both groups in the current study received training. In the absence of training, strength and performance often decline in the preoperative period [9, 14, 15], which was not the case in the current study. The standard training group in the present study underwent aerobic training (walking and cycling), balance training, and training of activities of daily life, such as chair rises and basic step training. In many other studies no exercise is prescribed during the preoperative period for a control group [9–15]. Because both groups trained, this may not only have prevented the decline as is seen in many other studies during the preoperative phase, but it also seems to suggest that the exact content of the training program is less relevant during a short preoperative phase. This finding is in line with the results of a recent study in which a control group improved walking and stair climbing after 6 weeks of nonspecific upper-body strength training [33]. There are no indications in the present study that additional heavy resistance training is superior to a program of more general aerobic training including some functional (strength demanding) tasks.

### 4.3. Post-Surgery Effects

The recovery of voluntary torque, stair climb, and walking ability at T4 was comparable to two earlier studies [34, 35], but somewhat lower than reported by others [14, 32]. There was a significant interaction (*P* = 0.043) between group and time for maximal torque of the knee flexors from T2 to T3. This interaction was probably not caused by the intensive strength training, because no interaction was present before surgery, and the preoperative training program was primarily focused on the knee extensors. Therefore, we consider this to be a sporadic finding. There were no other significant interactions between group and time after surgery.

### 4.4. Voluntary Activation

Before surgery, there were no differences in voluntary activation between the surgical and nonsurgical leg. As stated before, the lack of changes might be caused by an earlier or a future TKA of the nonsurgical leg. There were also no changes in voluntary activation after training and after surgery. The absence of changes in voluntary activation is not in line with two earlier studies [3, 36] that measured lower activation 4 weeks after surgery, but in accordance with two other studies in which no changes were found 12 weeks after surgery [37, 38]. The different findings regarding changes in voluntary activation may be explained by differences in timing of the measurements after surgery among studies. Thirty three months after surgery, significant increases in voluntary activation have been observed compared to before surgery [31]. Voluntary activation may decrease the first weeks after surgery and improve on a longer term.

### 4.5. Relationships between Quadriceps Strength and Physical Performance

The relationships between strength and physical performance and the observation that relationships are stronger later after surgery are in line with other studies [6, 32]. This may indicate that knee extension strength is an important factor for performance, especially in later stages of recovery. Consequently, postoperative strength training may improve functional recovery, which is in line with earlier research [2].

### 4.6. Clinical Relevance and Limitations

A major strength of the current study compared to other studies is that preoperative training had a relative high intensity and loads were progressively increased. For patients, the results of the present study indicate that it is unnecessary to subject patients to intensive training before TKA. A limitation is the low sample size in this study. Especially when studying postoperative effects, a larger sample size would be needed. It is, however, unlikely that preoperative strength training would be effective to promote recovery *after *surgery compared to standard preoperative training, because neither significant effects nor trends for superior effects of strength training were observed *before* surgery. Another limitation of the present study is a lack of randomization and the lack of blinding for therapists and patients.

## 5. Conclusion

We conclude that intensive strength training is feasible for the majority of the patients awaiting TKA. There were no indications that this intensive strength training is more effective than a standard training with respect to maximal knee extensor strength, voluntary activation, and performance in functional tests.

## Figures and Tables

**Table 1:** Exercises, sets and repetitions for the strength training group.

	Week 1	Week 2	Week 3	Week 4	Week 5	Week 6
Leg press 1-leg	3 × 15	3 × 12	4 × 12	3 × 10	4 × 10	4 × 8
Step up 1-leg	3 × 15	3 × 12	4 × 12	3 × 10	4 × 10	4 × 8
Squat	3 × 15	3 × 12	4 × 12	3 × 10	4 × 10	4 × 8
Leg extension 1-leg	3 × 15	3 × 12	4 × 12	3 × 10	4 × 10	4 × 8

**Table 2:** Characteristics of patients of the two training groups and drop-outs.

	Strength training (*N* = 10)	Standard training (*N* = 8)	Drop-outs (*N* = 4)	*P*
Sex (men/women)^a^	7/3	4/4	1/3	0.30
Age (years)^b^	71.8 (7.5)	69.5 (7.1)	73.3 (3.4)	0.33
BMI (kg/m^2^)^b^	27.9 (4.6)	27.9 (3.1)	26.3 (2.1)	0.71

^a^Differences tested using *χ* ^2^ test.

^b^Presented as mean (standard deviation), differences tested using Kruskall-Wallis Test.

**Table 3:** Strength measures, functional tasks, and WOMAC scores before (T1, T2) and after (T3, T4) surgery.

			T1 (*N* = 10/8)		T2 (*N* = 10/8)		T3 (*N* = 10/7)		T4 (*N* = 9/7)
MVT extension (Nm)	Affected side	STR	106 ± 45		111 ± 50	∗	63 ± 30	∗	76 ± 34
		STAND	121 ± 52		121 ± 50		70 ± 35		97 ± 40
	Unaffected side	STR	116 ± 47		123 ± 47		116 ± 44		118 ± 43
		STAND	137 ± 59		139±57		128 ± 65		138 ± 56

Doublet Torque (Nm)	Affected side	STR	49 ± 13		50 ± 16	∗	34 ± 10	∗	39 ± 12
		STAND	51 ± 19		48 ± 17		35 ± 13		39 ± 14
	Unaffected side	STR	53 ± 12		52 ± 14		50 ± 14		51 ± 16
		STAND	50 ± 15		50 ± 16		50 ± 17		50 ± 13

VA (%)	Affected side	STR	79 ± 13		78 ± 15		79 ± 9		80 ± 10
		STAND	80 ± 13		85 ± 8		84 ± 4		90 ± 8
	Unaffected side	STR	75 ± 19		78 ± 15		80 ± 13		83 ± 11
		STAND	84 ± 12		85±10		88 ± 6		91 ± 6

MVT flexion (Nm)	Affected side	STR	40 ± 22		43 ± 19	†	37 ± 18	∗	42 ± 17
		STAND	46 ± 25		50±24		36 ± 16		50 ± 23
	Unaffected side	STR	43 ± 29		47 ± 26		47 ± 27		47 ± 26
		STAND	57 ± 33		55 ± 30		55 ± 30		55 ± 26

Chair stand test (s)		STR	12.6 ± 2.6	∗	11.3 ± 2.1		13.3 ± 3.4	∗	11.8 ± 1.8
		STAND	12.3 ± 2.7		11.4 ± 1.8		12.5 ± 2.5		10.8 ± 1.5

Stair climb test (s)		STR	12.4 ± 3.1		11.6 ± 3.4	∗	20.9 ± 10.8	∗	12.8 ± 3.4
		STAND	12.9 ± 3.8		12.4 ± 3.3		17.6 ± 7.5		14.1 ± 0

6MWT (m)		STR	453 ± 81	∗	471 ± 92		380 ± 109	∗	456 ± 62
		STAND	460 ± 52		493 ± 55		440 ± 87		513 ± 97

WOMAC score (points)		STR	64 ± 11		65 ± 20		70 ± 16	∗	83 ± 15
		STAND	67 ± 11		67 ± 8		79 ± 11		93 ± 4

MVT: Maximal voluntary torque; VA: voluntary activation; 6MWT: six-minute walk test; WOMAC: McMaster Universities Osteoarthritis Index; STR: strength training group; STAND: standard training group. The numbers of patients in the intervention and standard training groups are displayed at the different times. Values represent mean ± standard deviation. *Significantly different compared to previous measurement for both groups combined (*P* < 0.05). ^†^Significant difference for groups between T3 and T2 (*P* = 0.043).

**Table 4:** Pearson correlation coefficients between maximal knee extension strength and functional tests.

		Chair stand test	Stair climb test	6-minute walk test
Affected side	T1	−0.03	−0.53*	0.41
	T2	−0.32	−0.58*	0.50*
	T3	−0.56*	−0.68*	0.76*
	T4	−0.56*	−0.74*	0.86*

Unaffected side	T1	−0.17	−0.59*	0.46
	T2	−0.32	−0.64*	0.54*
	T3	−0.47	−0.59*	0.66*
	T4	−0.52*	−0.73*	0.77*

*P < 0.05.
